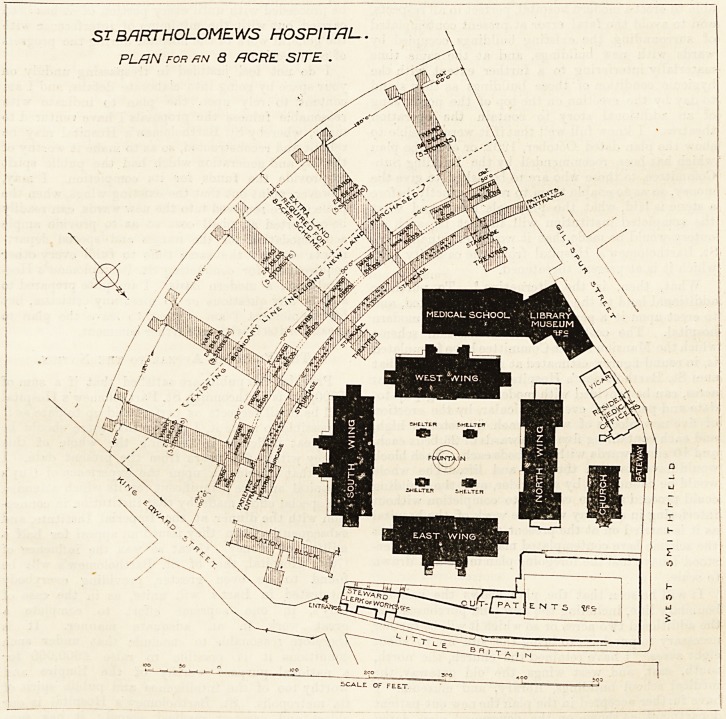# St. Bartholomew's Hospital and the Nation

**Published:** 1903-10-31

**Authors:** Henry Burdett


### HOSPITAL ADMINISTRATION.

### CONSTRUCTION AND ECONOMICS.

### ST. BARTHOLOMEW'S HOSPITAL AND THE NATION.

By Sir Henry Burdett.

It is stated in well-informed quarters that the lay authorities of St. Bartholomew's Hospital have it in contemplation to shortly issue an appeal to the nation for a large sum of money to reconstruct the hospital buildings at a cost in round figures of about £350,000. A meeting of the governors of St. Bartholomew's Hospital is announced for Thursday, the 5th November, and it is desirable that the public as well as the governors should realise the perils which underlie the adoption of the report of the Mansion House Committee in its present limited form. I will deal with the claims of St. Bartholomew's Hospital upon the nation a little later, but I may here point out that if the nation is to be appealed to successfully it can only be in support of a scheme which will provide St. Bartholomew's Hospital with a group of buildings perfect and up-to-date in every respect, where the most stringent hygienic requirements have been exactly fulfilled. If the appeal be based upon a plan which fulfils these essential conditions, in my judgment the amount of the capital sum asked for is a relatively minor consideration.

In an article which appeared in the *Times* on January 8th last the following accurate summary of the position is given: "St. Bartholomew's Hospital is antiquated and inefficient. In order to bring it up-to-date the authorities have bought a piece of land for a quarter of a million. To erect the required buildings and make the proposed alterations they must appeal to the public for a very large sum of money. But when the scheme is complete it will still be inadequate, and will become more so every year. The problem of renovating the hospital must be faced. Surely it would be wise to face it fully and do the thing well. The additional piece of land has been found insufficient fer the purpose. More is required, and it would be better to recognise this at once in calculating the sum to be asked for than to lose an opportunity which is not likely to recur."

Since that article appeared the Lord Mayor has presided over a special committee which was appointed in January last to inquire into the plans of the governors for the extension of the hospital on the present site, and the alternative suggestion for its removal. The report of this committee of the Lord Mayor was published in the *Times* of July 28th last, but it was not accompanied by any plans, nor by the full evidence given before it, a copy of which in its entirety ought to be sent to every governor and the press. It further does not contain any statement indicating fully and exactly the modifications which are contemplated in the plan dated October, 1902, of the proposed additions as originally propounded by the authorities of the hospital. The report merely stated that the Building Sub-Committee had before them three plans. That ultimately adopted unanimously will cost more than the amount of the original estimate, and, including the cost of alterations in the east, west and south wings, if it should be decided to remodel them, the contemplated cost would appear to be not less than £350,000. It would further appear from the report that three sets of plans were prepared by the architects, showing alternative schemes, all involving the removal of the church. The first provided for the demolition of all the present buildings, with the exception of the medical school block, and the erection of an entirely new hospital. The second plan proposed to leave standing, in addition to the school block, the east, south and west wings of the present quadrangle, and to erect on the remaining area the new buildings required. On the third plan all four of the wings of the present quadrangle are left standing. The Building Sub Committee unanimously came to the conclusion that the plan last referred to, which involves the retention of the quadrangle, is to be preferred. That plan provides for new buildings, including (1) a new out-patient department, dispensary, surgery wards (10 beds), hospital kitchens, etc.; (2) operation theatres on top of the north wing; (3) nurses' home; (4) new pavilion wards (68 beds) and chapel; (5) isolation pavilion (seven beds); (6) new pavilion wards (102 beds); (7) pathological department, mortuary, etc.; (8) quarters for resident staff and other officials; (9) heating and ventilating apparatus; (10) subways; (11) cost of alterations in the east, west and south wings.

The plan recommended by the Building Sub-Committee will therefore leave the east, south, and west wings, in which the wards are at present situated, as the main buildings in which patients will still be treated. Of course it is proposed to remodel these wings, but that after all will be but to tinker, because patients have been received into these buildings for about 150 years, and as Professor Sims Woodhead has pointed out, in regard to an old hospital building of about the same period, hospitals built at the date of St. Bartholomew's contain micro-organisms and dust-traps which find no place in the modern hospital. It is the simple truth that no scheme of reconstruction can effectually provide for their removal, and in consequence these micro-organisms must continue to exist to the danger of the patients in the wards. The wards of St. Bartholomew's have the still further disadvantage that they are back to back, that is, they do not provide the requisites for modern treatment in these days, when the open-air system has proved so efficacious. As I have before stated in the *Times*, it would be a grave responsibility for any medical staff, or any body of governors, to make permanent within the metropolis of the Empire, in connection with the only completely endowed hospital which we possess, the continuous occupancy by sick and injured persons of buildings which are altogether out of date, in which it is impossible to provide the essentials of air and light, free from micro-organisms, which we know to be most material to speedy and permanent recovery. Should, however, the governors of St. Bartholomew's Hospital fulfil the intention at present imputed to them of issuing an appeal for hundreds of thousands of pounds to perpetuate upon the nation this grave scandal, as it will undoubtedly prove to be, I make bold to say that they will find it impossible to fulfil their intention, because in such a case few if any who have it in their power to respond liberally to such an appeal will ever be induced to subscribe their money for such a purpose.

Since I first began to raise money for hospitals, nearly forty years ago, the average amount of individual gifts, and the number of givers have immeasurably increased. This satisfactory result is due to the quickening intelligence of the nation in regard to hospital matters, but for that very reason I rejoice to know that large sums of money are never likely to be given again in support of any proposal which is not up-to-date, and which does not fulfil to the utmost the hygienic and other requirements which the development of science has proved to be essential for the successful treatment of disease in buildings devoted to the purpose. What then is the present position? The committee have decided that it is essential that St. Bartholomew's Hospital shall be retained upon its present site. They require a hospital of at least 700 beds, and they wish to retain upon the site all the additional buildings necessary to fulfil the requirements of the greatest and oldest medical school. The present site, with the additional land purchased from Christ's Hospital, comprises an area of a little over six acres, and it is a fact that that area would be small indeed if it had but to contain all the buildings required for the purposes of a modern hospital with 700 beds. But when to these requirements we add everything essential to the successful conduct of a great medical school the inadequacy of the site must be admitted by everybody. It follows that now that the committee has determined to retain St. Bartholomew's Hospital on the

Smithfield site, it is essential that further land should be purchased from the governors of Christ's Hospital, so that the whole site available for the purposes of the hospital shall not be less than from 8 to 8¼ acres. The cost of this additional land, estimated at the rate already paid to Christ's Hospital, will be about £250,000. The first essential step which the governors have to take, therefore, is to purchase this further land, for then they will be able to erect a modern hospital, perfect in all respects, and to avoid the fatal error at present contemplated of surrounding the existing buildings occupied by wards with new buildings, and at the same time materially interfering to a further extent with the hygienic condition of those buildings as they stand to-day by the erection on the top of the north wing of an additional story to contain the operation theatres. I know full well that if it were possible to show the plan dated October, 1902, or even the plan which has been recommended by the Building Sub-Committee, to those who are to be asked to give the money, so as to enable them to realise exactly, before a stone is laid, what the proposals mean, and what the completed institution will in fact be, such an outcry would be made that it would effectually save St. Bartholomew's Hospital from the calamity with which it is at present threatened.

What, then, is the alternative? To purchase additional land to the extent I have indicated, and to erect upon it a perfected, up-to-date, and modern hospital. The cost of the reconstruction scheme which the Mansion House Committee have formulated is, in round figures, estimated at £350,000. For that sum St. Bartholomew's Hospital, with a site of eight acres, can be provided with modern buildings, up-to-date and perfect in every particular, by the erection of five new blocks of wards, each five stories high, and each containing five large wards of 26 beds each, and 10 small wards with two beds each. Each block would have its own theatres and lift. The whole would be connected by a corridor, and the building could proceed and be carried to completion without interfering in any way with the work of the hospital as it is carried on at the present time. In order that the scheme here contemplated may be readily understood I have had the foregoing plan made and drawn to scale.

It will be seen that the plan shows the existing boundary line, including the new land purchased and the additional two acres or so which it will be further necessary to purchase to bring the site up to about eight acres. I have retained the church, the north, south, east, and west wings, the old gateway, the medical school buildings, library, and museum. I have further accepted in the plan the new out-patient department, as shown on the governors' plan, dated October, 1902, the new buildings for the resident medical officers and the vicar, the steward, clerk of the works, etc. I have also added a new isolation department. I have further adopted the proposal in the plan of October, 1902, to clear the site at present occupied by the existing out-patients' and isolation blocks, the mortuary, the post-mortem, laboratories, and dispensary buildings, and in addition I propose to remove the chemical theatre. One of the great difficulties in dealing with the present site is the circumstance that it is possible that high buildings may be erected on that portion of the Christ's Hospital site not purchased by St. Bartholomew's Hospital. It will be seen that the new ward buildings on my plan are so placed as to reduce the objections arising from this possibility to a minimum, whilst they give each of the wards the maximum of sun, air, and light. On this plan it will be possible to gradually erect the new ward buildings so as to make those first erected available for the accommodation of the departments displaced. Thus the whole scheme might be proceeded with until every portion of it has been carried out with the minimum of interference with the general work of the hospital during the progress of each section.

I do not feel justified in trespassing unduly on your space by going into elaborate details, and I am content to rely upon the plan to indicate with reasonable fulness the proposals I have ventured to make, whereby St. Bartholomew's Hospital may be rebuilt and reconstructed, so as to make it worthy of the day and generation which had the public spirit to provide the funds for its completion. I may, however, point out that the existing wings, when the patients are removed into the new wards, can readily be converted at small cost so as to provide ample accommodation for the nurses and special departments and at the same time to fulfil every other requirement for conducting St. Bartholomew's Hospital upon a modern basis. I am quite prepared to answer any questions or to meet any criticism, but for the moment I am content to leave the plan to speak for itself without further comment.

### The Proposed Appeal to the Nation.

Provided the public are satisfied that if a sum of £600,000 is forthcoming, St. Bartholomew's Hospital will be in all respects as perfect and up-to-date as it is possible to make it, I believe that there should be no great difficulty in securing the whole of this money within five years from the present date. I base that conclusion upon the experience of Guy's Hospital and the magnificent results which attended the special effort made by that institution in connection with the dinner at the Imperial Institute, and subsequently when they issued an appeal for half a million of money. Great as was the influence of Guy's Hospital, that of St. Bartholomew's will be found to be even greater, providing everybody interested in Bart's. will unite, as in the case of Guy's, in one supreme effort to complete a great work in an adequate manner. It is therefore reasonable to conclude that under such conditions it is possible to raise £600,000 for a national purpose worthy of the Empire and worthy too of the intelligence and public spirit of its metropolis. St. Bartholomew's Hospital is our oldest and greatest medical school; it has sent forth from that school, and has at present representative medical men trained within its walls practising in London and the provinces, a vast army, equal to about one in nine of the whole of the 22,700 practitioners resident in England. Amongst Bartholomew's Hospital men 66 at present hold staff appointments in the general hospitals of London and 117 hold appointments in the hospitals of the provinces. Bartholomew's is worthily represented on the teaching staff of the Universities of Oxford and Cambridge, the Victoria University, University College, Liverpool, University College, Sheffield, the University at Birmingham, and the Bristol University College. Old students of this school are to be found at present serving on the staffs of at least twenty special hospitals in London. The list of honours obtained by students of St. Bartholomew's Hospital, and the names of those practising in the United Kingdom, Royal Navy, Royal Army Medical Corps, Indian Medical Service, or resident abroad, fill no fewer than 25 closely printed pages of St. Bartholomew's Hospital Journal for September. Collectively they number at least 3,250 distinguished medical men, including some of the most eminent and best-known members of the profession. I have merely summarised the figures, but a study of the names and a realisation of the enormous influence represented by those who make up this splendid total, must bring home with telling force the great debt of gratitude which the nation owes to St. Bartholomew's Hospital for its splendid services to the whole population in the past.

It is not a little remarkable that throughout the controversy which has raged around St. Bartholomew's Hospital during the last twelve months the voice of the medical staff has not made itself heard, and to this silence I attribute most of the mistakes and many of the dangers which at present threaten the future prosperity of our oldest and noblest hospital. It is due to the medical staff that at the meeting of Governors each Governor should be supplied with a copy of their report and recommendations, and that every member of the staff should be personally and individually invited to attend the Court of Governors, and to give expression to his views, so that any decision arrived at and any vote taken may be given with a full realisation of the problem in all its ramifications, and of the dangers which must attend the adoption of an imperfect or an inadequate scheme which can but lead to one result. To tinker, to temporise, to refuse to recognise plain facts, must end in disaster to the hospital, even supposing that the Governors were to have placed at their disposal to-morrow the £350,000 which they require to enable them to make certain temporary and inadequate changes in the existing buildings, and to add to those buildings on the present site new buildings to a certain extent. In such an event, granted that the whole plan indicated by the committee as it stands can be carried out to-morrow, it simply means that within the next quarter of a century it will be necessary—(1) To clear the site and build an entirely new hospital upon it, or (2) that a new site will have to be found, or (3) that the work of our greatest hospital will have to be carried on under conditions which will each year grow worse and worse until the hospital must necessarily forfeit its present great reputation, and place upon the shoulders of the next generation a task which may permanently cripple the usefulness of St. Bartholomew's Hospital for all time. If, however, an adequate scheme, including some such plan as that I have here ventured to submit, be adopted, which will really modernise the hospital and bring it up-to-date, the new St. Bartholomew's Hospital may stand as a monument to the intelligence and public spirit of the present generation. In such a case, but not otherwise, the nation may reasonably be relied upon to subscribe the £600,000 required for the work.

## Figures and Tables

**Figure f1:**